# Relationship Between Psychological Impacts of COVID-19 and Loneliness in Patients With Dementia: A Cross-Sectional Study From Iran

**DOI:** 10.3389/fpsyt.2022.814676

**Published:** 2022-04-06

**Authors:** Fatemeh Mohammadian, Mahya Rezaee, Arash Kalantar, Niayesh Mohebbi, Mahtab Motamed

**Affiliations:** ^1^Psychiatry Department, Tehran University of Medical Sciences, Tehran, Iran; ^2^Clinical Pharmacy Department, Tehran University of Medical Sciences, Tehran, Iran

**Keywords:** COVID-19, dementia, loneliness [source: MeSH], pandemic, psychological distress

## Abstract

**Objectives:**

Although the COVID-19 pandemic has affected people all around the world, the elderly is at a higher risk of suffering from its consequences. One of the serious concerns is developing loneliness and post-traumatic stress symptoms, which may contribute to cognitive decline at older ages. This study aimed to examine the psychological responses and loneliness in elderly patients diagnosed with dementia.

**Methods:**

Twenty-one patients diagnosed with dementia, with ages older than 40, and 19 caregivers were enrolled in the study. The patients have undergone a comprehensive neuropsychiatric interview and were assessed with De Jong Gierveld Scale for loneliness and Impact of Event Scale—Revised (IES-R). The severity of dementia was assessed by Functional Assessment Staging Tool (FAST Scale) and the Montreal Cognitive Assessment (MoCA).

**Results:**

No significant difference was seen in patients and caregivers in the IES-R and loneliness scales. A higher level of avoidance and social and total loneliness were seen in higher FAST levels (*p*-value: 0.046). There was a negatively significant correlation between MoCA score and avoidance. Hyperarousal was significantly correlated with emotional loneliness in patients.

**Conclusion:**

We found a direct relationship between cognitive decline and the psychological impacts of COVID-19. Our results highlight the need for more comprehensive studies to further investigate the influence of the pandemic on the worsening of cognitive impairment and loneliness in patients with dementia.

## Introduction

The COVID-19 pandemic has affected billions of people all around the world in many aspects. Besides the growing number of cases every day, people all over the world experience political, socio-economic, and psycho-social impacts (1). The spread of the virus has forced nations to develop policies such as lockdown protocols and physical or social distancing (2). While these regulations are crucial to halt the transmission of the coronavirus and help relieve the pressure on the healthcare system, social isolation leads to an increase in the prevalence of mental health problems such as depression (3), generalized anxiety disorders (4), insomnia (5), as well as feeling of loneliness among individuals (2).

Despite affecting all age groups, the older people are at a higher risk of suffering from prolonged social restrictions (6). Adults aging 65 and higher and anyone with pre-existing medical conditions are more likely to experience the complications from COVID-19 (7). Considering the elevated risk of developing a serious illness in these vulnerable groups of people, they need to limit their social contacts (8). One of the serious concerns is developing loneliness amid older people in this challenging time (9).

Loneliness is defined as a negative subjective feeling of deficient relationships and being alienated from others (10). It is the state of being unhappy with the quantity or quality of the current social attachments (11). In a study conducted in Sweden, loneliness was strongly correlated with an increased risk of all-cause mortality and developing chronic disorders in older adults, including hypertension, stroke, cardiovascular disease, and cognitive impairment (10). Loneliness is a key contributor to a wide range of mental health problems as well as poor physical health outcome (6). Older people are reported to have a higher level of loneliness as they have experiences of death of loved ones, retirement, and chronic illnesses (12).

According to some cultural beliefs in Iran as a traditional country, it is not socially and culturally accepted for an old person to be very outgoing, have multiple gathering with his or her own friends, making new friends, or start a romantic relationship. In this culture, a wise old person is pictured as a quiet and introvert one who is more occupied in deep thoughts. Regarding all these beliefs, talking and complaining about loneliness is highly stigmatized.

There exists proof that engaging in activities and social interactions are associated with better physical and mental health in older people (13).

Additionally, due to the complications of maintaining social relationships under the new COVID-19-related regulations, effort and attention should be directed toward the vulnerable groups, and they must be provided with social support (2).

Neurocognitive disorders, of which dementia is the major form, are a broad class of impairments in cognition, frequently in the older population. Due to the growth of the elderly population in the world, the number of patients with dementia is also increasing, which is mostly seen in developing countries where two thirds of patients with dementia live. There were 35.6 million people living with dementia worldwide in 2010, and this is estimated to increase to 65.7 million by 2030 and 115.4 million by 2050. The estimated prevalence of dementia in people aged 60 years and older is 8.7% in the Middle East and North Africa region—about 2.3 million people—and this is expected to rise to 4.4 million by 2030 (14).

Dementia interferes with cognitive function and performing activities of daily living. As a result, individuals may feel a sense of loss in their independence and a disruption to their sense of self. Evidence supports that social isolation and the lack of social integration may contribute to cognitive decline at older ages (15).

Patients with dementia have limited access to accurate information about COVID-19 and have problems remembering and following healthcare-related instructions, such as wearing a mask and personal hygiene, thus increasing their risk of infection. The safety and effectiveness of the COVID-19 vaccine and the specific side effects of the different types of vaccine in patients with dementia should be assessed comprehensively (16, 17).

On the other hand, people with dementia who lived in nursing homes are isolated from society and community because they could not use virtual communication tools. Obviously, the elderly in these centers lost face-to-face contact with family members in the COVID-19 crisis, which could lead to social isolation and loneliness (18).

Liu et al. demonstrated the negative impact of the COVID-19 pandemic crisis on patients with dementia and their caregivers. This review emphasized the negative burden of the pandemic on patients with dementia, which was categorized in six domains of well-being, including well preventing, well diagnosing, well treating, well supporting, well living, and well dying (19).

The COVID-19 crisis, as an infectious disease outbreak, could impact people's emotions in various aspects and thus could emphasize the emergent need for a novel questionnaire designed to assess behavioral changes and an increased level of anxiety. Riad et al. designed the COVID-19 Induced Anxiety Scale to evaluate the potential anxiety source in the general population, which could be utilized in the elderly population (20). In a study conducted in Poland, post-traumatic stress disorder (PTSD) symptoms related to COVID-19 worsened in both healthy people and patients diagnosed with depression and anxiety, and a lower emotional well-being was associated with a higher severity of PTSD (21).

Several studies demonstrated the relationship between post-traumatic stress symptoms and loneliness in adults. A recent study conducted in 2021 revealed that a longitudinal association between subtypes of loneliness and post-traumatic stress symptoms exists among older adults (22). Targeting the early psychiatric symptoms in response to a trauma, such as the COVID-19 pandemic, can ameliorate the feeling of loneliness.

This study aimed to examine the psychological responses and loneliness in older patients admitted to the dementia outpatient clinic of Roozbeh Hospital, Tehran, Iran, during the COVID-19 outbreak. We predict that patients with more severe dementia may suffer from loneliness due to higher PTSD symptoms related to COVID-19.

## Materials and Methods

### Participants

Patients with dementia who were diagnosed by a cognitive neurologist and referred to the dementia outpatient clinic were consecutively recruited to the study after obtaining their informed consent. This study was conducted between September 2020 and November 2020, about 6 months after the first peak of the COVID-19 pandemic in Tehran, Iran.

### Inclusion Criteria

The inclusion criteria included patients older than 40 years with a diagnosis of dementia syndromes, including Alzheimer's disease, vascular dementia, or combined dementia (Alzheimer's disease and vascular dementia), Lewy body dementia, and frontotemporal dementia based on the Diagnostic and Statistical Manual of Mental Disorders criteria as assessed by a neurologist.

### Exclusion Criteria

The exclusion criteria involved major psychiatric disorders, such as depression, schizophrenia, psychotic disorders causing rapidly progressing dementia, treatable causes of dementia such as metabolic disorders, history of severe liver, renal, and heart failure, and unwillingness to participate in the study at any time and for any reason.

### Procedure

For the first step, the patients' demographic characteristics, including age, marital status, place of residence, occupation, level of education, and history of psychiatric illness, were completed using the researcher-designed questionnaire. The patients have undergone a comprehensive neuropsychiatric interview held by an expert neurologist or psychiatrist. The researchers evaluated the patients and their caregivers for symptoms of COVID-19 infection in the last 3 months.

In the next step, the patients and caregivers were assessed for loneliness using the De Jong Gierveld Scale. The participants were then assessed for psychological reactions to COVID-19 with the Impact of Event Scale (IES-R) questionnaire. Since patients with mild and moderate dementia were included in this study, they had the necessary cooperation and cognitive capacities to complete the utilized questionnaires.

The patients' cognitive status was assessed using the Montreal Cognitive Assessment (MoCA) battery (23) and Functional Assessment Staging (FAST) scale (24).

### Instruments

#### The De Jong Gierveld Scale

This is an 11-item questionnaire which scores social loneliness (5 items) and emotional loneliness (6 items) and is validated in Farsi (25). The content validity of the questionnaire based on Waltz and Bausell's content validity index was acceptable (0.881), and it had appropriate internal consistency (α: 0.778). Hosseinabadi R et al. demonstrated that the Persian version of the 11-item De Jong Gierveld Scale had significant correlations with the Philadelphia Geriatric Center Morale Scale and also showed acceptable concurrent validity and reliability of this scale for measuring loneliness in Iranian older adults (25).

#### Impact of Event Scale Questionnaire

The questionnaire includes 22 questions with three subscales, including avoidance (8 questions), intrusive thoughts (8 questions), and hyperarousal symptoms (6 questions). Each question is answered on a Likert scale of 0 to 4. In patients scoring higher than 33, a diagnosis of post-traumatic stress disorder should be considered. The questionnaire is validated in Farsi. The Cronbach's alpha coefficients for the three subscales of the IES-R were high (ranging from 0.84 to 0.93), and the average inter-item correlation was between 0.42 and 0.62. Construct validity was evaluated using maximum likelihood exploratory factor analysis (MLEFA). In MLEFA, the Kaiser–Meyer–Olkin test value was 0.931 and Bartlett's test value was 6,022.415 (*p* < 0.001) (26).

#### The Montreal Cognitive Assessment

The MoCA test, developed by Nasr al-Din et al. for mild cognitive impairment (MCI) and dementia, evaluates the different domains of cognitive functioning (27). The reliability of this test was 92%, based on Cronbach's alpha, and its internal consistency was 83%. The maximum score of the test is 30, with a score of 26 or higher considered to be normal. This test, which is executed within 10 min, includes different domains: short-term memory (5 points); executive function, including Trail Making Test-B, Clock Drawing Test, and visuospatial function test (cube copying) (5 points); attention and working memory (6 points); language, including naming, repetition, and fluency (6 points); abstraction (similarity) (2 points); and orientation to time and place (6 points). Patients with scores of 26 or higher did not have any cognitive impairments (normal MoCA), whereas patients with scores lower than 26 probably had cognitive impairments (28). Rashedi et al. found a sensitivity of 94% and a specificity of 90% for MCI and Alzheimer's disease, with a cutoff score of 20 (23).

#### Functional Assessment Staging Tool

FAST is a reliable and valid assessment technique for evaluating functional deterioration in dementia patients throughout the entire course of the disease. The FAST scale is categorized into seven stages of dementia, in which stage 6 and above are correlated with a severe stage of dementia (24). The Persian version of FAST was evaluated by Noroozian et al. The area under the receiver operating characteristic curve was calculated as 0.952. It had a sensitivity of 92.2% and specificity of 98.0% for the differentiation of normal cognition from MCI and a sensitivity of 99.0% and specificity of 93.7% for the discrimination of subjects with Alzheimer's disease from MCI (29).

### Sample Size Calculation

The sample size in this study is calculated using the following formula (30, 31):

Sample size = $\frac{Z_{\left(1-\frac{\alpha }{2}\right)}^{2}\;/\;SD^{2}\;}{d^{2}}\approx \;41$

where α = 0.05,

SD = 1.64, and

*d* = 0.5.

### Statistical Analysis

The collected data were analyzed using IBM SPSS v.22 (IBM Corp., Armonk, NY, USA), and appropriate statistical tests were performed. The significance level was determined as *P* < 0.05.

### Ethical Considerations

Written informed consents were obtained from all subjects prior to enrollment in the study. The study protocol was approved by the local ethics review committee of Tehran University of Medical Sciences (ethical approval number: IR.TUMS.MEDICINE.REC.1400.611).

## Results

### Demographic and Clinical Characteristics of the Sample

Twenty-one patients and 19 caregivers were enrolled in the study. As shown in Table 1, no significant difference existed between caregivers and patients in terms of demographic characteristics.

**Table 1:** Demographic characteristics of patients and caregivers.

**Variable**	**Patients**	**Caregivers**	***P*** **-value**
Age (mean ± SD)	59.76 ± 9.71	56.57 ± 11.78	0.345
Gender (*N*) Female Male	9 12	10 9	0.536
Marital status Single Married	4 17	2 17	0.451
Education (*N*) Illiterate Primary High school diploma University	3 13 3 2	2 5 7 5	0.087
Working status (*N*) Unemployed Employed	14 7	11 8	0.567
Past psychiatric history (*N*) Positive Negative	10 11	9 10	0.987
History of COVID-19 in a family member (*N*)			0.121
Positive Negative	15 6	9 10	

*All the p-values are non-significant*.

The clinical characteristics of patients are reported in Table 2. The majority of our patients (*n* = 8) had a diagnosis of MCI with Alzheimer's disease, followed by those with mixed dementia (*n* = 5).

**Table 2:** Clinical characteristics of patients.

**Variable**	**Number**
Type of cognitive impairment MCI^a^ Alzheimer's dementia Vascular and mixed dementia Dementia of Lewy body Traumatic brain injury Parkinson's disease	8 1 2 3 5 1
FAST^b^ level 3 4 5	10 9 2
MoCA^c^ (mean ± SD)	22.4 ± 3.4

a *Mild cognitive impairment*.

b *Functional assessment staging tool*.

c *Montreal cognitive assessment*.

Almost half of the number of patients (*n* = 10) were at stage 3 in FAST, nine were at stage 4, and only two were at stage 5.

### IES-R Scores in Patients and Caregivers

We have examined the impact of COVID-19 in patients and their caregivers with the IES-R. The scores are reported in Table 3. No significant difference was seen in patients and caregivers in the IES-R scale. Thirteen patients and 12 caregivers scored above 24 in the IES-R, in whom a clinical diagnosis of PTSD should be further considered.

**Table 3:** Impact of event scores in patients and caregivers.

**Impact of events**	**Patients**	**Caregivers**	***P*** **-value**
Avoidance	12.38 ± 3.73	12.78 ± 3.22	0.713
Intrusive thoughts	7.04 ± 3.78	7.89 ± 4.50	0.526
Hyperarousal	9.90 ± 3.22	9.73 ± 5.49	0.908
Total score	29.33 ± 8.69	30.42 ± 10.44	0.724
Impact of events level <24 24–32 (clinical concern) 33–38 (probable diagnosis of PTSD^a^) >39	8 5 6 2	7 5 2 5	0.333

*All the p-values are non-significant*.

a *Post-traumatic stress disorder*.

### Loneliness Scores in Patients and Caregivers

Additionally, loneliness was scored in patients and caregivers. The detailed report of the loneliness scores can be found in Table 4. Most of the patients (*n* = 17) experienced a moderate level of loneliness, whereas three of them experienced severe loneliness. Similarly, 16 caregivers had a moderate level of loneliness, while only three of them experienced a severe level of loneliness.

**Table 4:** Loneliness scores in patients and caregivers.

	**Patients**	**Caregivers**	***P*** **-value**
Emotional Loneliness	3.52 ± 1.12	3.84 ± 1.38	0.433
Social loneliness	2.85 ± 1.01	2.57 ± 1.16	0.429
Total score	6.38 ± 1.68	6.43 ± 1.77	0.942
Loneliness level 1 2 3	1 17 3	0 16 3	0.627

*All the p-values are non-significant*.

### Relationship Between Demographic and Clinical Characteristics With IES-R Scores and Loneliness in Patients and Caregivers

There were no significant differences in the impact of events based on age, gender, and working and marital status in either the patients or the caregivers. No differences were seen in patients with and without a history of psychiatric disorders. The difference in IES-R scores was not significant between individuals whose family member had COVID-19 and those whose family member did not have COVID-19.

Similarly, we could not find any difference between loneliness scores based on age, gender, working and marital status, and past psychiatric history either in patients or in caregivers. The loneliness level was not different between individuals whose family member had COVID-19 and those whose family member did not have COVID-19.

### Relationship of Cognitive Impairment IES-R Scores and Loneliness in Patients

A higher level of avoidance was seen in higher FAST levels (*p*-value: 0.046) (Table 5). Higher social loneliness scores and total loneliness scores were seen in patients with FAST 5 in comparison with those with FAST 3 or 4 (Table 5). There was a negatively significant correlation between MoCA score and avoidance (*p***-**value: 0.046). No significant correlation was seen with total or subtypes of loneliness.

**Table 5:** Relationship of cognitive impairment with impact of event and loneliness scores.

	**FAST level**	**Mean ±SD**	***P*** **-value**
Avoidance	3.00	11.20, 2.52	0.046
	4.00	12.44, 4.24	
	5.00	18.00	
Intrusive thoughts	3.00	5.90, 3.03	0.138
	4.00	8.88, 4.28	
	5.00	4.5, 0.70	
Hyperarousal	3.00	8.80, 2.78	0.252
	4.00	10.55, 3.67	
	5.00	12.50, 0.70	
Impact of event score (total)	3.00	25.9, 6.24	0.209
	4.00	31.88, 10.72	
	5.00	35	
Emotional loneliness	3.00	3.20, 1.13	0.268
	4.00	3.66, 1.11	
	5.00	4.5, 0.71	
Social loneliness	3.00	2.40, 0.70	0.028
	4.00	3.00, 1.00	
	5.00	4.50, 0.50	
Loneliness (total)	3.00	5.60, 0.47	0.032
	4.00	6.67, 0.47	
	5.00	9.00 ± 0.0	

### Relationship of IES-R Scores and Loneliness Scores in Patients

For the next step, we examined the correlation of IES-R scores and loneliness scores in patients and caregivers. Hyperarousal was significantly correlated with emotional loneliness in patients, while in caregivers such a correlation was not seen.

The total score of IES was instead significantly correlated with emotional loneliness (*p*-value: 0.012) in caregivers. Besides this, there was a positive relationship between avoidance and total scores in loneliness in caregivers (*p*-value: 0.040).

## Discussion

In the present study, majority of patients and caregivers reported moderate levels of loneliness, and nearly half of patients and caregivers scored above 24 in the IES-R. Total and social loneliness was higher in patients with higher FAST stages. A higher level of avoidance was seen in patients with higher FAST stage and lower MoCA score.

Based on the pandemic management theory, individuals go through seven phases when facing a pandemic. It starts with an orientation phase, in which individuals assess the situation and their coping resources. This phase is followed with short adaptability. Consequently, acute and chronic phases of negative consequences occur, which are characterized by symptoms like anger, sadness, post-traumatic stress symptoms, and fatigue and which may lead to a full-blown illness. However, these phases could end up in positive consequences, such as a positive attitude toward life and empathic behavior. Maintaining affective communication with others, contact with one's identity, construction of a life sense, and a perception of wholeness are necessary to sustain a healthy identity during the pandemic (32). This illustrates that timely addressing post-traumatic stress symptoms and loneliness would prevent further negative consequences of COVID-19.

### Loneliness in Older Patients

According to studies from many countries like United States and Austria, older adults experienced loneliness following the COVID-19 pandemic (33–36). Seifert et al. demonstrated that older people's loneliness was increased in a sample of 1,990 older adults aged 65 to 95 after the implementation of physical distancing due to the Switzerland government's regulations (33). In contrast, there are some recent evidence from Austria or Germany suggesting that loneliness was not increased during the COVID-19 outbreak (34–36). According to a recent review, the adaptive ability of older people in the face of COVID-19 may vary based on social, cultural, and economic factors (37). In a study in German and Polish populations during the COVID-19 pandemic, older people were found to have a higher level of quality of life, wellbeing, and life satisfaction and a lower level of anxiety. The authors associated this to higher education, financial stability, and limited access to news (38). This underscores the need for such investigations in different countries worldwide as well as in developing countries like Iran.

The mediators of developing loneliness in older people are not well studied. Women, individuals living alone with no children and with lower income, and individuals who are unsatisfied with their contact with neighbors are reported to be at a greater risk of loneliness (33). In our study, majority of patients and caregivers reported moderate levels of loneliness, although our findings showed no difference in patients and their caregiver in terms of the level of loneliness. This may be due to the collective culture of Iran, where older people are supported by their children and grandchildren, protecting them from feeling lonely. It should also be noted that there might be stigmas toward expressing loneliness, and feeling lonely may be translated to being weak or not having strong religious beliefs. As a result, loneliness may be underestimated or masked.

### Avoidance and Loneliness

We found a positive relationship between cognitive decline and loneliness. The COVID-19 pandemic has posed numerous risks to people with dementia (39). Patients with dementia were influenced by the COVID-19 pandemic through various ways, including difficulties in remembering and following new emerging guidelines, deprivation from a cognitively enriched environment, increased feeling of anxiety as well as depression, and sense of loneliness (40–42).

Apart from the above-mentioned impacts of COVID-19, it should be noted that, as dementia progresses, experiencing some levels of loneliness is due to decreased participation in social activities and less social engagement.

One might also speculate that individuals with cognitive decline are not able to develop more effective and active mechanisms of facing a trauma.

According to our findings, patients with more cognitive decline are more likely to present avoidance as a response to the stress related to the COVID-19 pandemic. Such defense mechanisms may underlie the increased loneliness in people with a deeper cognitive decline.

This can be seen as a two-way relationship, where avoidance may theoretically result in increased loneliness and cognitive decline, on one hand, and loneliness worsens avoidance and cognitive decline in the other. Although we have found a direct correlation between loneliness and avoidance in caregivers in this study, such correlation was not significant in the patients.

Jun Holwerda et al. showed that, after risk factor adjustment, older populations with a feeling of loneliness were more likely to develop dementia, so the prevention of feeling lonely may protect the vulnerable older population from cognitive decline both in the group of patients with cognitive impairment and their caregivers with currently intact cognition status (43).

Right after the occurrence of a stressful event and before the emergence of avoidance response can be considered as a golden time for monitoring for stress responses in patients with dementia and utilizing appropriate psychotherapeutic interventions to withhold the cascade which otherwise may ultimately end up in cognitive decline and loneliness.

### Hyperarousal and Loneliness

Emotional loneliness was positively correlated with hyperarousal in patients. According to the hypervigilance hypothesis of loneliness, lonely individuals show higher previgilance for social threats (44). Meng et al. reported a positive correlation between loneliness and alertness in college students in China, where a feeling of fear played a mediating role (45). In a study by Layden et al., loneliness was associated with increased resting state functional connectivity between several nodes involved in tonic alertness (46). In line with these findings, it can be assumed that the higher hyperarousal response to COVID-19 in our patients have led to greater feelings of loneliness.

### Strengths and Limitations

Utilizing the IES-R questionnaire and the De Jong Gierveld Scale, we could assess the effect of COVID-19 in patients with dementia in different aspects. To the best of our knowledge, this is the first study to examine the relationship between loneliness, IES-R, and cognitive decline in COVID-19.

The most important limitation of this study was the small sample size due to limited access to patients and their caregivers during the COVID-19 pandemic, considering that tele-visits are not widely used in our country and not easily utilized by the older people.

Another study limitation is the heterogenicity of our patients in terms of the type of dementia, which included MCI, Alzheimer's disease, mixed vascular dementia, and Lewy body dementia. According to the diverse characteristics of each dementia syndrome, various behavioral and psychiatric reactions to a specific stressful event could be expected.

Filling out the questionnaires would be difficult for some patients with cognitive decline. However, it is better to use simpler questionnaires with the cooperation of the patients' caregivers in the case of more severe stages of cognitive disorders.

The cross-sectional notion of our study prevents us to elaborate a causal relationship between loneliness and the COVID-19 pandemic. Moreover, we have not explored the loneliness feeling in dementia patients or their caregivers before the COVID-19 pandemic, so we could not determine the temporal effect of the pandemic on loneliness severity. Therefore, we utilized the IES-R and, in correlation to loneliness, De Jong Gierveld Scale to be able to indirectly investigate the effect of the COVID-19 pandemic as a stressful event on the loneliness of dementia patients and their caregivers.

Similarly, it was not possible to determine the effect of the pandemic on the exacerbation or acceleration of cognitive impairment in patients with dementia.

## Conclusion

This study was a pilot observational study that evaluates the effect of the COVID-19 pandemic on the aggravation of loneliness in patients with dementia and their caregivers. Our results highlight the need for more comprehensive studies to further investigate the influence of the pandemic on the worsening of cognitive impairment and loneliness in patients with dementia.

## Data Availability Statement

The raw data supporting the conclusions of this article will be made available by the authors, without undue reservation.

## Ethics Statement

The studies involving human participants were reviewed and approved by Tehran University of Medical Sciences. The patients/participants provided their written informed consent to participate in this study.

## Author Contributions

FM: 30% (study design, drafting, analyzing the data, and data monitoring). MR and AK: 15% (collecting the data and documentation and reviewing the manuscript). NM: 10% (study design and reviewing the manuscript). MM: 30% (study design, drafting, analyzing the data, and data monitoring). All authors contributed to the article and approved the submitted version.

## Conflict of Interest

The authors declare that the research was conducted in the absence of any commercial or financial relationships that could be construed as a potential conflict of interest.

## Publisher's Note

All claims expressed in this article are solely those of the authors and do not necessarily represent those of their affiliated organizations, or those of the publisher, the editors and the reviewers. Any product that may be evaluated in this article, or claim that may be made by its manufacturer, is not guaranteed or endorsed by the publisher.
